# Simultaneous production of laccase and degradation of bisphenol A with *Trametes versicolor* cultivated on agricultural wastes

**DOI:** 10.1007/s00449-017-1783-1

**Published:** 2017-05-23

**Authors:** Shengquan Zeng, Jie Zhao, Liming Xia

**Affiliations:** Key Laboratory of Biomass Chemical Engineering of Ministry of Education, College of Chemical and Biological Engineering, Zhejiang University, Hangzhou, 310027 China

**Keywords:** Bisphenol A, Laccase, Solid state fermentation, Agricultural wastes, *Trametes versicolor*

## Abstract

Solid state fermentation with *Trametes versicolor* was carried out on agricultural wastes containing bisphenol A (BPA). It was found that BPA degradation was along with the occurrence of laccase production, and wheat bran and corn straw were identified as suitable mixed substrates for laccase production. In the process of BPA degradation with *T. versicolor*, laccase activity increased rapidly at the 6th–10th day after inoculation. Moreover, BPA can enhance the production of laccase. After 10 days of fermentation, degradation rate of BPA exceeded 90% without the usage of mediators ABTS and acetosyringone at pH 4.0–8.0. In addition, metal ions did not affect the BPA degradation with *T. versicolor*. In vitro, the optimum pH range of BPA degradation with laccase was in the acidic region with the optimal performance of pH 5.0. Metal ions Cu^2+^, Zn^2+^, and Co^2+^ showed little effect on BPA degradation. However, Fe^3+^ and Fe^2+^ substantially inhibited the BPA degradation. Natural mediator acetosyringone showed optimum enhancement on BPA degradation. Greater than 90% of the estrogenic activity of BPA was removed by *T. versicolor* and its laccase. Compared to in vitro degradation with laccase, this study shows that the process of simultaneous laccase production and BPA degradation with *T. versicolor* was more advantageous since BPA can enhance the laccase production, mediators were unnecessary, degradation rate was not affected by metal ions, and the applicable pH range was broader. This study concludes that *T. versicolor* and laccase have great potential to treat industrial wastewater containing BPA.

## Introduction

Laccases (benzenediol: oxygen oxidoreductase, EC 1.10.3.2) belong to the family of multicopper oxidases and can catalyze the oxidation of various phenolic compounds and aromatic amines with concomitant four-electron reduction of molecular oxygen to water [1]. Laccases are known to be specific, ecologically sustainable, and proficient catalysts; they have been applied in many fields, such as food-processing industry [2], dye decolorization [3], paper and pulp industry [4], and bioremediation [5–7]. Some bacterial laccases have been found and well characterized [8]; however, laccase is mostly produced by the white-rot fungi with lignin-degrading ability [9, 10]. *Trametes versicolor* belongs to white-rot basidiomycetes and has received much research attention because its laccase exerts the highest redox potential among other laccases [11].

However, the high cost and low production of laccases have become the main limitations for their extensive industrial applications. Thus, the major research interest on laccase production is focused on the discovery of a highly effective production system. Solid state fermentation (SSF) is a fermentation process occurring in the absence or near absence of free liquids, in which microorganisms grow on solid materials [12]. SSF provides several advantages in enzyme production over submerged fermentation (SF): lower energy expenditure, lower wastewater output, higher concentration of products, and less expensive downstream processing [13]. Moreover, this method of fermentation can be a suitable process to utilize agricultural and industrial cellulosic wastes as solid substrates, which offers potential economic advantages and helps in solving environmental pollution problems caused by the disposal of these wastes. Various lignocellulosic wastes have been used as solid substrates in the SSF of laccase [14–17]. *T. versicolor* laccase is particularly promising for industrial applications of environmental remediation due to its high catalytic activity on various types of contaminants [18, 19]. There are some reports on industrial–agricultural wastes like wheat ran [20], tomato pomace [21], horticultural waste [11], and olive leaves [22] for laccase production in SSF with *T. versicolor*. However, corn straw and wheat bran have not been tested as mixed substrates for laccase production by *T. versicolor*.

Bisphenol A (BPA) is used as an intermediate to produce polycarbonate and epoxy resins [23]. BPA is an endocrine-disrupting chemical with potential detrimental effects on human health, including sperm-count reduction, breast and prostate cancer, early sexual maturation in females, and immunodeficiency [24]. Therefore, the pollution of BPA into the environment can result in fearful environmental concerns. In view of its application to target a wide spectrum of pollutants, biological degradation of aromatic pollutants has attracted the attention of environmentalists. The lignin modifying living organisms and their enzymes have shown great potential to target a wide range of aromatic compounds [25, 26]. Several fungal laccases have shown the potential of BPA degradation; such fungi include *Trametes polyzona* [27], *T. versicolor* [19], and *Grifola frondosa* [28]. The presence of mediators, which can extend the range of laccases to various substrates, makes laccases attractive for increasing the number of targeted pollutants [19]. Hence, the laccase-mediator systems can enhance the conversion of BPA [29].

In this work, the process of simultaneous laccase production and BPA degradation with *T. versicolor* cultivated on agricultural wastes was thoroughly investigated. Effects of pH, mediators and metal ions on BPA degradation with *T. versicolor* and laccase were tested. There are some reports about BPA degradation with *T. versicolor*; in these papers, BPA was degraded by *T. versicolor* cultivated in the liquid medium. However, in this study, simultaneous BPA degradation and laccase production was realized in the solid state fermentation with *T. versicolor* cultivated on agricultural wastes, and this is a economical and environmentally friendly process. Moreover, this study represents for the first time particular comparison of BPA degradation with white rot fungi and their laccases from the point of pH, mediators, metal ions, and laccase production.

## Materials and methods

### Chemicals and solid substrates

Bisphenol A, vanillin, acetosyringone, 2,2′-azino-bis(3-ethylbenzothiazoline-6-sulfonic acid) (ABTS) and 1-hydroxy-benzotriazole (HBT) were purchased from Sigma-Aldrich (USA). All other chemicals were of analytical grade.

Corn straw, corncob, rice straw, sawdust, bagasse, and wheat bran, which were obtained from local farms, were air dried and milled to pass through a 20-mesh screen.

### Microorganism and inoculum preparation

*Trametes versicolor* ZJ-02, which was screened from the rotten wood and stored in the Laboratory of Biochemical Engineering of Zhejiang University, was used for laccase production. The strain was cultured on potato dextrose agar slants at 30 °C for 7 days and stored at 4 °C. The mycelia from the agar slants were transferred to 250 mL Erlenmeyer flasks containing 50 mL of the following inoculum medium (g/L): glucose, 10.0; yeast extract, 1.0; (NH_4_)_2_SO_4_, 1.4; KH_2_PO_4_, 2.0; CaCl_2_, 0.3; urea, 0.3; and MgSO_4_·7H_2_O, 0.1. The medium was adjusted to pH 4.8. The culture was allowed to grow in the above medium under aerobic conditions at 30 °C for 7 days and used as inoculums.

### Laccase production

The medium for laccase production had the following composition (%, dry weight basis): agricultural wastes, 56; wheat bran, 40; (NH_4_)_2_SO_4_, 2.5; CaCl_2_, 0.48; KH_2_PO_4_, 0.5; MgSO_4_·7H_2_O, 0.5; and CuSO_4_, 0.02. The initial pH value of the medium was adjusted to 5.0 and the water content of it was 65%. After autoclaved at 121 °C for 60 min, 10% inoculums were inoculated into 20 g of solid substrate in a 500 mL Erlenmeyer flasks and incubated at 30 °C for 10 days. Effect of the types of agricultural wastes (corn straw, corncob, rice straw, sawdust, and bagasse) on laccase production was investigated. All assays were independently performed in duplicates, and the data shown correspond to mean values with standard deviation.

### Laccase assays

Laccase was extracted by adding 10 volumes of distilled water to the fermented substrate, and shaking at 180 rpm for 1 h at 30 °C. The mixture was centrifuged at 4000 rpm for 20 min at 4 °C to remove the mycelium. The supernatant was collected and used for enzyme activity assay.

Laccase activity was determined using ABTS as substrate [30]. The reaction mixture included 50 µL of culture supernatant, 950 µL of citrate–phosphate buffer (pH 4.5), and 1 mL of ABTS solution (2 mM). The temperature was adjusted to 30 °C. Oxidation was followed by increasing absorbance at 420 nm (*ε* = 3.6 × 10^4^/cm/M). One activity unit was defined as the amount of enzyme that oxidized 1 µM of ABTS per minute. The activities were expressed in unit per gram of dry substrate (U/g ds).

### Simultaneous production of laccase and degradation of bisphenol A with *T. versicolor*

Solid state fermentation with *T. versicolor* was carried out on agricultural wastes containing bisphenol A (25 mg/kg solid substrate). The inoculation and incubation procedures were the same as that introduced above. The cultivation time was set as 10 days. A control test with the same concentration of BPA without the addition of *T. versicolor* was performed. Effects of pH (3.0–8.0), mediators (HBT, ABTS, vanillin and acetosyringone; 0.1–1 mmol/kg solid substrate), metal ions (Fe^2+^, Fe^3+^, Cu^2+^, Co^2+^ and Zn^2+^; 10 mmol/kg solid substrate) were investigated, respectively. After 10 days of fermentation, the residual BPA on cultures was extracted by a mixture of methanol and water (1:1) (1/10, W/V) and shaken at 200 rpm for 4 h and then centrifuged at 4000 rpm for 15 min. The supernatant was collected for measuring the residual BPA concentration. The BPA concentrations of all samples were measured by an HPLC system (Agilent 1200, USA) fitted with an Agilent Eclipse XDB-C18 column (150 mm × 4.6 mm × 5 µm) and eluted with 60% acetonitrile in water at a flow rate of 1.0 mL/min at 30 °C. The injection volume was 20 µL and the running time of samples was 5 min. The BPA concentrations of all samples were determined by reference to a standard curve of BPA detected at 228 nm.

All experiments were performed in duplicates, and the degradation degree was calculated by the equation $\frac{{(C_{0} - C)}}{{C_{0} }} \times 100\%$, where *C*_0_ is the initial concentration, and *C* is the final concentration.

### Degradation of bisphenol A by laccase

The laccase-catalyzed degradation of BPA was conducted in a 50 mL Erlenmeyer flask, which contained 10 mL of the reaction mixtures. The reaction mixtures contained sodium citrate buffer (100 mM and pH 5.0) with initial laccase activity of 25 U/L and BPA of 25 mg/L. The bisphenol A concentration (25 mg/L) was set according to the previous detection of the wastewater of four plastics plants in our lab, which showed the bisphenol A concentration of 10–25 mg/L. A control test with the same amount of heated-denatured laccases and BPA concentration in 100 mM sodium citrate buffer (pH 5.0) was performed. Both degradation and control tests were kept at 30 °C under aerobic condition by stirring at 120 rpm. The effects of parameters in this process on degradation, such as pH (3.0–8.0), mediators (HBT, ABTS, vanillin and acetosyringone), and metal ions (Fe^2+^, Fe^3+^, Cu^2+^, Co^2+^ and Zn^2+^) were investigated, respectively.

Before measuring the BPA concentration, the enzyme reaction was halted by adding a small amount of concentrated acetic acid to the reaction mixtures to reduce the pH to approximately 2.0. The BPA concentrations of acidified samples were measured and degradation rate was calculated using the formula introduced above.

### Estrogenic activities of bisphenol A treated with *T. versicolor* and laccase

Estrogenic activity test using the human breast estrogen-sensitive MCF7 cells was performed according to the method described previously, which was called the E-screen test [31]. The test uses MCF7 cells and compares the cell yield achieved after 6 days of culture in medium supplemented with 5% charcoal-dextran stripped human serum in the presence of bisphenol A treated with *T. versicolor* and laccase or untreated. The estrogenic activities of samples were assessed by determining the proliferative efficiency (PE), which is the ratio between the cell number in the presence and in the absence of samples (control).

## Results and discussion

### Effects of the types of agricultural wastes on laccase production

**Fig. 1:**
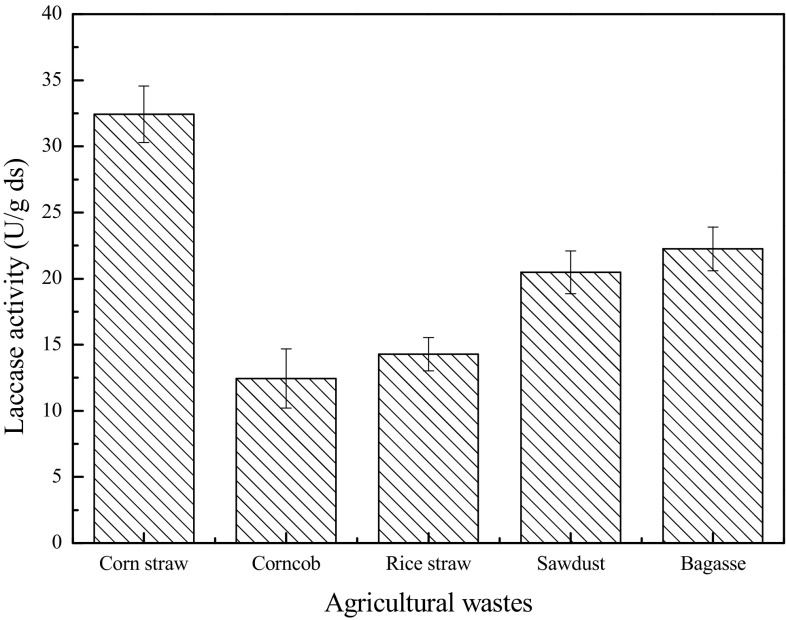
Effect of the types of agricultural wastes on laccase production by *T. versicolor* under solid state fermentation. Fermentation conditions include initial moisture content 65%, initial pH 5, and temperature 30 °C

The selection of an appropriate substrate is important for the success of the SSF process. The production of laccase by supplementing various agricultural wastes in the presence of wheat bran was evaluated. Figure 1 shows that among the substrates tested, wheat bran supplemented with corn straw exhibited the highest laccase activity (32.09 U/g ds), followed by bagasse (22.25 U/g ds). The minimum activity was achieved by corncob (12.45 U/g ds). The microbial product yield varied with different types of substrates [32, 33]. This finding was confirmed by the current results, which indicated that the laccase activity varied with different types of substrates. Compared to previous reports, the laccase activity level achieved in this study with *T. versicolor* was proved significant. By using horticultural waste as substrate for *T. versicolor* SSF, Xin and Geng have found a maximum laccase activity value of about 8.6 U/g substrate within 7 days of fermentation [11]. Sarnthima et al. used the agro-industrial wastes to produce laccase by the white-rot fungus *Lentinus polychrous* Lév under SSF. A high laccase activity of 4.4 U/g substrate was yielded using rice bran as a sole substrate after 21 days of cultivation, and the highest laccase activity of 10.0 U/g substrate was produced using the mixed substrates of rice bran and rice husk (2:1 by weight) after 17 days of culture [34]. Sathishkumar et al. used banana peel and wheat bran as mixed substrates to produce laccase, and its production was optimized by response surface methodology, which resulted in the highest activity of 7.96 U/g in the optimized medium [35]. The present study has demonstrated the utility of wheat bran and corn straw, which were readily available as raw materials, as mixed substrates to produce laccase by SSF.

### The time course of simultaneous laccase production and BPA degradation

**Fig. 2:**
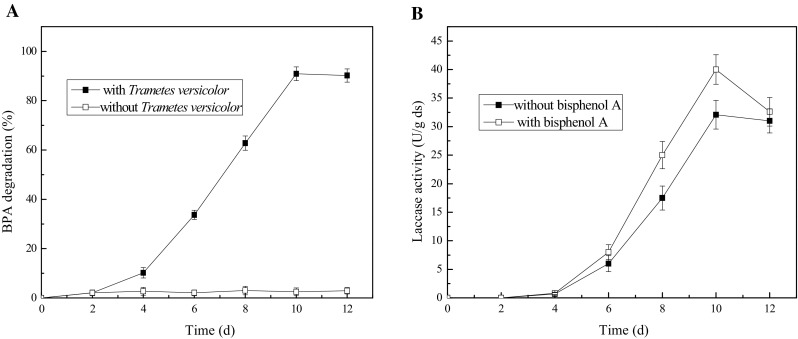
BPA degradation (**a**) and laccase production (**b**) with *T. versicolor*. Fermentation conditions include initial moisture content 65%, initial pH 5 and with wheat bran and corn straw as substrates

**Fig. 3:**
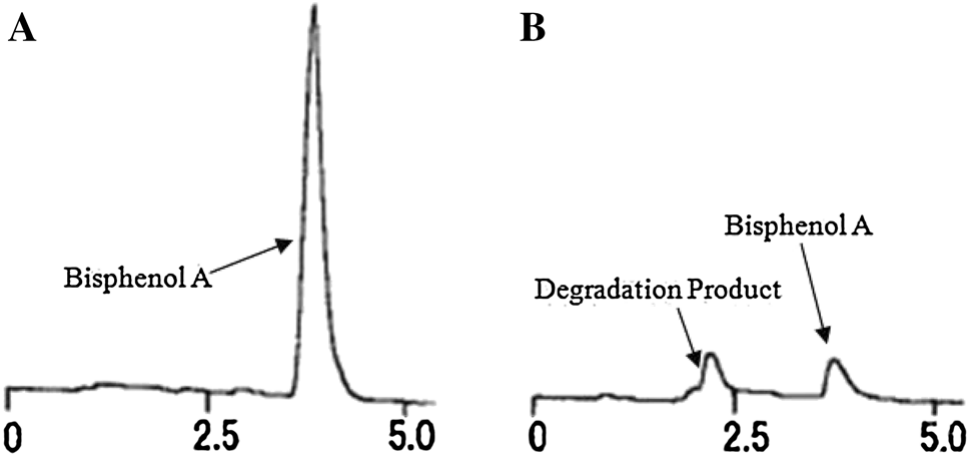
The HPLC chromatogram of BPA degradation with *T. versicolor*. Culture time: **a** 0 days; **b** 10 days

BPA was added to the solid state fermentation medium with a final concentration of 25 mg/kg solid substrate. Figures 2a and 3 show that BPA was degraded about 10% in 4 days and 90% after 10 days of fermentation by *T. versicolor*. Figure 2b indicates that laccase activity increased rapidly at the 6th–10th day after inoculation, in parallel to the BPA degradation. Thus, the degradation of BPA was closely related to the process of laccase production. The maximum laccase activity was observed at the 10th day after inoculation and at this time the maximum BPA degradation rate was also achieved. Therefore, simultaneous production of laccase and degradation of BPA was achieved by *T. versicolor* cultivated on agricultural wastes. Tsioulpas et al. also reported that high laccase activity was measured in the growth medium while 69–76% phenolic compounds was removed by *Pleurotus* spp. [36]. In the present study, Fig. 2b also shows that laccase activity was enhanced by the addition of BPA. This result concurs with a previous study that the lignin modifying enzyme activities were stimulated by BPA concentrations up to 300 mg/L [37].

### Effect of pH on BPA degradation

**Fig. 4:**
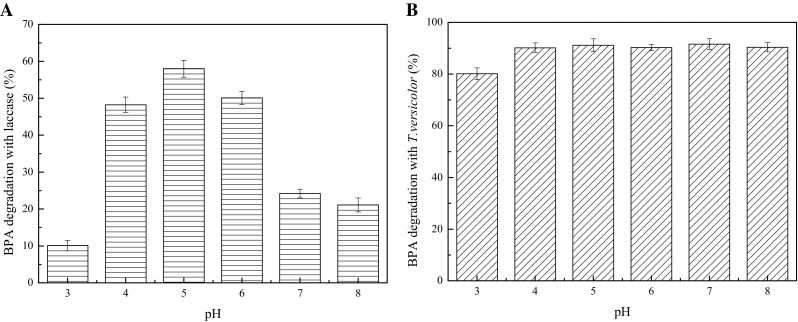
Effects of pH on BPA degradation with laccase (**a**) and *T. versicolor* (**b**). Fermentation conditions with *T. versicolor*: temperature 30 °C, moisture content 65%, with a reaction time of 10 days. Reaction conditions with laccase: 120 rpm, 25 U/L, 25 mg/L BPA, 30 °C, with a reaction time of 1 h

An enzyme should demonstrate its catalytic action on a target substrate under various environment conditions to achieve effective practical application. The pH value of a reaction process can influence the enzyme activity and the rate of interaction between enzymes and substrates [19]. Figure 4a shows that the optimum pH range of BPA degradation with laccase from *T. versicolor* was in the acidic region with the optimal performance of pH 5.0. This result agrees with the previous report indicating that most fungal laccases optimally operate in acidic pH [38]. However, Chhaya and Gupte [24] reported that the optimum pH was 6.0 when laccase from *Fusarium incarnatum* was utilized to remove BPA. Therefore, the optimal pH value for BPA degradation by laccase was species specific.

The pH of the medium is of much significance in the process of SSF because the pH affects microbial growth and metabolic activity. The culture pH also strongly influences many enzymatic processes and the transport of various components across the cell membrane [39]. To study the effect of pH on the BPA degradation during the SSF process with *T. versicolor*, BPA was degraded by *T. versicolor* in the pH range of 3.0–8.0 which is shown in Fig. 4b. The degradation rate of BPA exceeded 80% and there is no significant difference in pH value ranging from 4.0 to 8.0. These results are consistent with previous reports which demonstrated that decolorization ratios of aniline blue and indigo carmine were not affected by pH value in the process of dye decolorization with *T. versicolor* [40]. Therefore, compared to the reaction in vitro with laccase, it can be concluded that BPA degradation in the SSF process of *T. versicolor* was applicable in a broader pH range and was favorable to the practical applications of industrial wastewater treatment.

### Effect of metal ions on BPA degradation

**Fig. 5:**
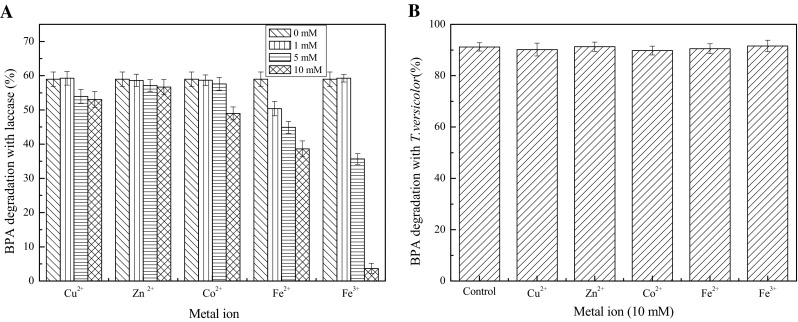
Effect of metal ions on BPA degradation with laccase (**a**) and *T. versicolor* (**b**). Fermentation conditions with *T. versicolor*: temperature 30 °C, moisture content 65%, with a reaction time of 10 days. Reaction conditions with laccase: 30 °C, pH 5.0, 120 rpm, 25 U/L, 25 mg/L BPA, with a reaction time of 1 h

Different metal ions are often found in industrial wastewater. Metal ions can change enzyme activity due to their ability to stabilize or destabilize the protein. Thus, the effects of the presence of such metal ions on the process of BPA degradation should be determined. Figure 5a indicates that 10 mM of Cu^2+^, Zn^2+^ and Co^2+^ showed little effect on the degradation rate of BPA, whereas Fe^2+^ and Fe^3+^ substantially inhibited the BPA degradation. The electron transport system of laccase can be hindered by iron ions, thereby inhibiting the substrate conversion [41]. However, all metal ions at the concentration of 1 mM did not exert a significant effect on BPA degradation. Moreover, Kim and Nicell [19] also reported that Cu^2+^, Zn^2+^, and Co^2+^ at concentrations lower than 1 mM showed no significant effect on the BPA degradation by laccase.

However, compared to in vitro degradation with laccase, as showed in Fig. 5b, 10 mM metal ions (Cu^2+^, Zn^2+^, Co^2+^, Fe^2+^ and Fe^3+^) did not markedly affect the BPA degradation in the SSF process with *T. versicolor*. This mitigates the limitation of metal ions of high concentration in industrial or agricultural wastewater. Baldrian reported that white-rot fungi can adsorb and accumulate metal ions [42]. This can explain that BPA degradation rate was not significantly affected by the addition of metal ions.

### Effect of mediators on BPA degradation

**Fig. 6:**
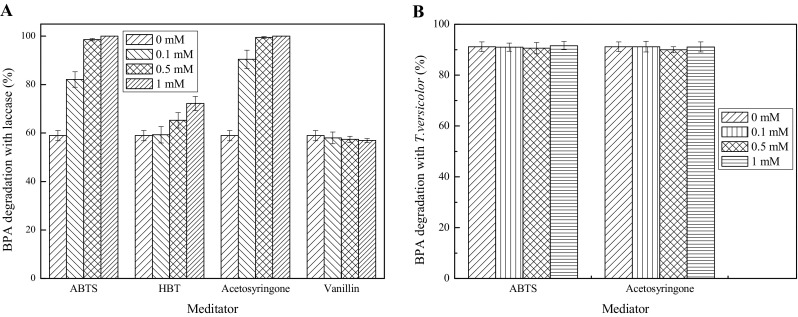
Effect of mediators on BPA degradation with laccase (**a**) and *T. versicolor* (**b**). Fermentation conditions with *T. versicolor*: temperature 30 °C, moisture content 65%, with a reaction time of 10 days. Reaction conditions with laccase: 30 °C, pH 5.0, 120 rpm, 25 U/L, 25 mg/L BPA, with a reaction time of 1 h

Mediators are small molecules that can act as electron-transfer intermediates between enzymes and substrates. Hence, mediators can enhance the conversion of substrates. Suitable mediators should be discovered to explore the potential of laccases as bioremediation agents. The current study investigated the effects of synthetic mediators ABTS and HBT on BPA degradation with laccase. Figure 6a shows that ABTS effectively improved the degradation rate of BPA, which varied from 58.94% (without ABTS) to 82.11% (0.1 mM ABTS). The concentration of ABTS increased to 0.5 mM and allowed the laccase to achieve up to 98% BPA degradation. Compared with ABTS, HBT showed lesser positive effect on BPA degradation with only 72.20% degradation rate of BPA with the addition of 1 mM HBT. The results indicated that ABTS was a better laccase-mediator system than HBT for BPA degradation, and this finding agreed with the report of Kim and Nicell [19]. ABTS follows the electron-transfer mechanism, that is, an electron is obtained from the substrate rather than a hydrogen atom, which is the case with HBT adopting the transfer mechanism of hydrogen atoms [43, 44]. Thus, the electron-donating substituents in the aromatic ring of BPA can enhance the susceptibility of BPA to oxidation by using a laccase-ABTS system.

However, the major constraints for application at an industrial scale of synthetic mediators (ABTS and HBT) include high cost and toxic properties [45]. Therefore, an increased interest is seen in the utilization of cheaper and eco-friendly natural mediators, which can be obtained from renewable sources and plants [46]. The effects of natural mediators, namely, vanillin and acetosyringone, on BPA degradation were tested in the present study. Vanillin has been reported as a mediator to decolorize dyes showing promising results [47]. However, in the present study, the data (Fig. 6a) showed that vanillin exerted a slightly negative effect on BPA degradation. This finding might be relevant to the different types of substrates with various structural features. Acetosyringone (0.1 mM) showed 90.41% conversion of BPA within 1 h in the presence of 25 U/L laccase, which was higher than that of ABTS (0.1 mM). However, a 4-h period or 50 U/L laccase is required to obtain the same conversion in the absence of acetosyringone (data not showed). The results indicated that the addition of mediators might require low reaction time and enzyme dosage, and the nature of the mediator plays a considerable role in BPA degradation by using a laccase-mediator system. The role of the natural mediator acetosyringone in enhancing BPA conversion has not been cited in the literature. Thus, the novelty of the present study increased.

The effects of laccase mediators ABTS and acetosyringone on the degradation of BPA was tested in the SSF process with *T.* *versicolor.* Compared to in vitro degradation with laccase, Fig. 6b indicates that both mediators ABTS and acetosyringone showed no effect on BPA degradation in the SSF process with *T. versicolor*. Therefore, laccase mediators are unnecessary for BPA degradation in the SSF process with *T. versicolor*. Mediators, including natural and synthetic mediators, can serve as an electron shuttle between laccase and target compounds, extending the range of laccase to different substrates [19]. Natural mediators involved in the natural degradation of lignin by white-hot fungi can be derived from oxidised lignin units or directly from fungal metabolism [48]. In the present study, degradation rate of BPA was virtually not affected by the concentrations of mediators (ABTS and acetosyringone). Therefore, it is assumed that some compounds produced from degradation of the lignin component of wheat bran and corn straw might perform as mediators.

### Estrogenic activities of BPA treated with *T. versicolor* and laccase

It is not uncommon for compounds to be degraded into toxic metabolites, therefore the estrogenic activities of the endocrine disrupting chemical BPA before and after treatment by the *T. versicolor* and laccase were investigated. MCF7 cells are estrogen-sensitive and the endocrine disrupting chemical BPA can induce the cell proliferation. As showed in Table 1, the proliferative efficiency of MCF7 cells treated with the BPA sample degraded by laccase was similar to that by *T. versicolor*, both of which were close to the control. Therefore, according to the reduction in proliferative efficiency, greater than 90% of the estrogenic activity of BPA was removed by *T. versicolor* and laccase. Moreover, Cajthaml et al. also reported that BPA was efficiently degraded by *T. versicolor* in liquid media and *T. versicolor* nearly removed the estrogenic activity of BPA within 7 d [49].

**Table 1:** Estrogenic activities of BPA treated with *T. versicolor* and laccase

Conditions	Control	BPA (25 mg/L)	BPA (25 mg/L) + laccase (25 U/L)^b^	BPA (25 mg/kg substrate) + *T.* *versicolor* ^c^
PE^a^	1	5.37 ± 0.18	1.39 ± 0.12	1.36 ± 0.15

^a^PE (proliferative efficiency) is the ratio between the cell number in the presence and in the absence of samples (control)

^b^The reaction time of BPA degraded by laccase was 24 h

^c^The culture time of BPA degraded by *T. versicolor* was 10 days

## Conclusions

The results showed that wheat bran and corn straw can be effectively used as mixed substrates for the SSF process with *T. versicolor* to produce high activity laccase. The effects of reaction conditions on BPA degradation in the SSF process with *T. versicolor* and in vitro with laccase were elucidated. It was demonstrated that the SSF process by *T. versicolor* had great potential to be utilized for simultaneous laccase production and BPA degradation. Moreover, greater than 90% of the estrogenic activity of BPA was removed by *T. versicolor* and laccase. In terms of pH, mediators, metal ions and laccase production, BPA degradation in the SSF process with *T. versicolor* was more superior than in in vitro degradation with laccase. Further studies on identification of degradation products and degradation mechanism are recommended.
